# Effects of CuO nanoparticles on *Lemna minor*

**DOI:** 10.1186/s40529-016-0118-x

**Published:** 2016-01-27

**Authors:** Guanling Song, Wenhua Hou, Yuan Gao, Yan Wang, Lin Lin, Zhiwei Zhang, Qiang Niu, Rulin Ma, Lati Mu, Haixia Wang

**Affiliations:** 1 School of Medicine, Shihezi University, Shihezi, 832000 China; 2 Shanghai Key Laboratory of Atmospheric Particle Pollution Prevention (LAP3), Shanghai, 200433 China; 3 Research Center of Water Pollution Control Technology, Chinese Research Academy of Environment Sciences, Beijing, 100012 China; 4 Department of Life Science, Shandong University of Technology, Zibo, 255049 China

## Abstract

**Background:**

Copper dioxide nanoparticles (NPs), which is a kind of important and widely used metal oxide NP, eventually reaches a water body through wastewater and urban runoff. Ecotoxicological studies of this kind of NPs effects on hydrophyte are very limited at present. *Lemna* *minor* was exposed to media with different concentrations of CuO NPs, bulk CuO, and two times concentration of Cu^2+^ released from CuO NPs in culture media. The changes in plant growth, chlorophyll content, antioxidant defense enzyme activities [i.e., peroxidase (POD), catalase (CAT), superoxide dismutase (SOD) activities], and malondialdehyde (MDA) content were measured in the present study. The particle size of CuO NPs and the zeta potential of CuO NPs and bulk CuO in the culture media were also analyzed to complementally evaluate their toxicity on duckweed.

**Result:**

Results showed that CuO NPs inhibited the plant growth at lower concentration than bulk CuO. *L. minor* roots were easily broken in CuO NPs media under the experimental condition, and the inhibition occurred only partly because CuO NPs released Cu^2+^ in the culture media. The POD, SOD, and CAT activities of *L.* *minor* increased when the plants were exposed to CuO NPs, bulk CuO NPs and two times the concentration of Cu^2+^ released from CuO NPs in culture media, but the increase of these enzymes were the highest in CuO NPs media among the three kinds of materials. The MDA content was significantly increased compared with that of the control from 50 mg L^−1^ CuO NP concentration in culture media.

**Conclusion:**

CuO NPs has more toxicity on *L.* *minor* compared with that of bulk CuO, and the inhibition occurred only partly because released Cu^2+^ in the culture media. The plant accumulated more reactive oxygen species in the CuO NP media than in the same concentration of bulk CuO. The plant cell encountered serious damage when the CuO NP concentration reached 50 mg L^−1^ in culture media. The toxicology of CuO NP on hydrophytes must be considered because that hydrophytes are the basic of aquatic ecosystem.

## Background

Nano-technology has a strong claim to be regarded as the first important advance in technology of the third millennium (Robert 2012). Given the rapid development of nanotechnology, an increasing risk of human and environmental exposure to nanotechnology-based materials is apparent. However, data on the potential environmental effects of nanoparticles (NPs) are scarce (Clément et al. 2013; Zhang et al. 2013), particularly on the effects and mechanisms of these NPs on higher plants (Nair et al. 2010; Song et al. 2012; Miralles et al. 2012).

Metal oxide NPs are manufactured at a large scale for both industrial and household use (Aitken et al. 2006; Xia et al. 2013). CuO NPs, an important kind of metal oxide NPs, are used in catalysis, gas sensors, solar energy conversion, high-temperature superconductors, and field-emission emitters (Chowdhuri et al. 2004; Yin et al. 2005; Dar et al. 2008; Jammi et al. 2009). With such large-scale applications, CuO NPs will inevitably reach bodies of water through waste water and urban runoff. Therefore, understanding the risks of this kind of NPs to aquatic ecosystems is essential. The toxicity study of CuO NPs on aquatic organisms has drawn considerable attention in recent years. Aquatic creatures, such as fish, algae, bacteria, and crustaceans, are adversely affected by CuO NPs (Kahru and Dubourguier 2010; Gunawan et al. 2011; Zhao et al. 2011; Li et al. 2012). However, the toxic effects of CuO NPs on hydrophytes are scare at present. Aquatic macrophytes are important for oxygen production, nutrient cycling, water quality control, sediment stabilization, and shelter for aquatic organisms and wildlife (Mohan and Hosetti 1999); these plants are vital in maintaining the stability of aquatic ecosystems. Thus, the toxic effect of CuO NPs on aquatic plants should be studied on time.

*Lemna minor*, a duckweed species, is a widespread, free-floating aquatic macrophyte. *L. minor* is a food source for waterfowl and a shelter for small aquatic invertebrates. *L. minor* grows fast and reproduces more rapidly than other vascular plants. Because of these characteristics, duckweed is often used in water body restoration and ecotoxicological studies (Song et al. 2012; Žaltauskaitė and Norvilaitė 2013). To study the toxicity effect of CuO NPs on *L. minor*, the macro growth and microphysical response of *L. minor* exposed to CuO NPs in several concentrations were investigated compared with those of *L. minor* exposed to bulk CuO and soluble Cu^2+^. These physical indexes include the peroxidase (POD), catalase (CAT), and superoxide dismutase (SOD) activities of this floating plant, as well as its malondialdehyde (MDA) and chlorophyll contents, under different treatments.

## Methods

### Plant materials, growth conditions, and treatment procedures

*L. minor* was collected from the region of Shandong Province in China (N 36°48.183′, E 117°55.528′). The plant was stored in glass troughs (0.5 m × 0.8 m × 0.6 m) and illuminated with metal halide lamps (72 µmol m^−2^ s^−1^) as pre-treatment during a daily photoperiod of approximately 16 h for 1 month before the experiments. The pretreatment medium was 1/10 modified Steinberg medium (Michael and Hans-toni 2002) (Table 1) with a pump for circulation, and the culture was maintained at 26 ± 2 °C. The medium was renewed with the same concentration of modified Steinberg medium every two weeks.

**Table 1:** Composition of the modified Steinberg medium

Substance	Concentration (mg L^−^1)
KNO_3_	350
KH_2_PO_4_	90
K_2_HPO_4_	12.6
MgSO_4_ 7H_2_O	100
Ca(NO_3_)_2_ 4_2_HO	295
MnCl_2_ 4H_2_O	0.18
H_3_BO_3_	0.12
Na_2_MoO_4_	0.044
ZnSO_4_ 7H_2_O	0.18
FeCl_3_ 6H_2_O	0.76
Na_2_EDTA 2H_2_O	1.5

### Characterization of CuO NPs and bulk CuO

CuO NPs and bulk CuO were purchased from Shanghai Jingchun Reagent Limited Company, China. CuO NPs, with the purity is greater than 99.5 %, particle diameter is 40 nm and a surface area is 25–40 m^2^ g^−1^.The purity of bulk CuO is more than 99.9 %, particle diameter is 10 μm. The morphology of the CuO NPs was examined by transmission electron microscopy (TEM, JEOL 100CX, Japan). The particle diameter of CuO NPs in solution and the zeta potential of CuO NPs and bulk CuO in solution were measured using a 90 plus particle size analyzer (DR-525, Brookhave Instruments Corporation, USA) at 12 h after media preparation. The Cu^2+^ concentration that the nano-CuO released to the culture media was measured by ICP-MS 7500ce, Agilent, USA at 24 h after media preparation.

### Test design

The experiment media were divided into three treatments. The media for treatment 1 consisted of 1/10 modified Steinberg medium with 0, 10, 50, 100, 150, and 200 mg L^−1^ CuO NPs, which were ultrasonicated for 30 min. The media for treatment two consisted of 1/10 modified Steinberg medium with 0, 10, 50, 100, 150, and 200 mg L^−1^ bulk CuO. The media for treatment three were 1/10 modified Steinberg medium with an amount of CuCl_2_ that supplied twice the Cu^2+^ concentration released from CuO NPs in treatment 1 media. The tests were performed in 500 mL beakers containing 200 mL media. The pH of all of the culture media was adjusted to 6.5.

Before the experiments, *L. minor* was disinfected by immersing in NaClO (1 %, v/v) for 3 to 5 min, and then rinsing with distilled water for three times. Culture media were renewed every 2 days. The cultured *L.* *minor* was divided into two groups with different treatments. Each treatments contain three replicates. The inoculum of the first group included 12 fronds (only plant with two or three fronds were selected), which were used in determining the number of fronds, root length, and fresh weights at 96 h. The inoculum of the other group included 1 g of fronds (measured after 5 min of blotting on dry tissue paper), which were used in determining the chlorophyll, POD, CAT, SOD, and MDA at 96 h. The plants in all of the groups and treatments were randomly placed together in a growth chamber with 60 % humidity at 28 °C in the light (36 µmol m^−2^ s^−1^) and at 26 °C in the dark. The phytotoxicity experiment lasted for 4 days. The suspensions were stirred using a glass rod every 8 h. The change in the number of fronds is calculated according to the following formula (Tkalec et al. 1998; Song et al. 2006).

$${\text{The change of frond number }} = \frac{{{\text{no}} . {\text{ of fronds at day n}} - {\text{ no}} . {\text{ of fronds at day 0}}}}{{{\text{no}} . {\text{ of fronds at day 0}}}}$$

### Enzyme extraction and chlorophyll determination

To obtain the enzyme extract, 500 mg of whole plant was homogenized in 5 mL cold potassium phosphate buffer (0.1 M, pH 7.8). The homogenate was centrifuged at 15,000*g* (4 °C) for 15 min in a refrigerant centrifuge. The supernatant was used as the enzyme extract. The enzyme extraction was conducted at 4 °C.

Chlorophyll content was measured using the duckweed fronds. The fronds were whetted and distilled in ethanol (96 %), and then the extracts were measured spectrophotometrically at 665, 649, and 470 nm (Zhao 2000a).

### Enzyme assays

For POD, the mixture consisted of 50 mM potassium phosphate buffer (pH 7.0), 1 mL; 0.2 % H_2_O_2_, 2 mL; 0.2 % guaiacol, 0.95 mL; and enzyme extract, 50 µL. The enzyme activity was measured by monitoring the increase in absorbance at 470 nm during polymerization of guaiacol into tetraguaiacol. CAT activity was measured spectrophotometrically by following the consumption of H_2_O_2_ at 240 nm (Liu 2006). SOD was measured using SOD detection kit that was produced in Nanjing Jiancheng Bioengineering Institute. Assay was carried out according to the specification of the detection kit. MDA activity was measured according to Zhao (2000b). The enzyme extracts of 1.5 and 2.5 mL thiobarbituric acid (TBA, 0.5 %) were boiled for 20 min, and then centrifuged. Afterwards, the supernatant was measured spectrophotometrically at 532, 600, and 450 nm.

### Statistical analysis

Results were expressed as mean ± standard deviation (SD). The obtained data were evaluated by Student’s *t* test compared with their corresponding control (0 mg L^−1^ CuO) using Prism 5.0 statistical package. The statistical significance was considered at p < 0.05.

## Results and discussions

### Characterization of CuO NPs and the particles in media

**Fig. 1:**
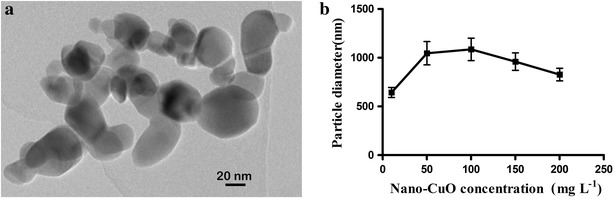
TEM observation of CuO NPs (**a**) and size of CuO NPs aggregates in culture media (**b**)

The TEM image of CuO NPs and the particle size of the NPs in the culture media were showed in Fig. 1. The CuO NPs were near sphere shaped, and the diameter of individual NPs were basically less than 40 nm.

The CuO NPs aggregated to form larger sizes in the culture media. The aggregations are likely driven by the divalent ions and low zeta potential (Griffitt et al. 2007). The divalent ion effect can be due to NP bridging via ionic bonds to form NP–*M* ^+^–NP (where *M* ^+^ is the salt cation), with Na^+^ in the culture media, thereby promoting NP aggregation (Wang et al. 2011a). In addition, divalent cations such as Mg^2+^ and Ca^2+^ (also present in the culture media) have been shown to induce NP aggregation (Akaighe et al. 2012). Agglomerates forming a neck between two or more particles create an area of negative surface curvature, and nucleation occurs at this interface under equilibrium conditions. This action can result in the fusion of the agglomerates and a reduction in total particle surface area (Chang et al. 2012).

**Fig. 2:**
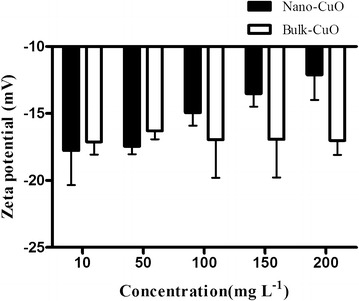
Changes in the zeta potentials of CuO NPs and bulk CuO in the culture media

The zeta potentials of CuO NPs and bulk CuO in culture media are shown in Fig. 2. The zeta potentials of both nano- and bulk CuO were negatively charged in culture media. The zeta potentials of CuO NPs increased with the increase of NP concentration in media. The zeta potential absolute values of bulk CuO were higher than those of CuO NPs in the same concentration. The value of the zeta potential can be related to the stability of colloidal dispersion. Zeta potential indicates the degree of repulsion between adjacent, similarly charged particles in the dispersion. A high absolute value of the zeta potential of the particles in a solution indicates that the solution or dispersion has a high capacity to resist aggregation. When the zeta potential absolute value of the particles in a solution is low, attraction exceeds repulsion, and then the dispersion breaks and flocculates. The general dividing line between stable and unstable suspensions is generally considered at either +30 or −30 mV. Particles with zeta potentials that are more positive than +30 mV or more negative than −30 mV are normally considered stable (Duman and Tunc 2009). The results of the present study indicate that the colloidal dispersion of these culture media with CuO NPs decreases when the NPs concentration is increased, and the stability of culture media-added bulk CuO was higher than that of culture media added with the same concentration CuO NPs. The particle diameter decreased with the increase in CuO NP concentration when the CuO NPs concentration was higher than 100 mg/L, which can be the reason of the zeta potential changes in these culture media.

**Fig. 3:**
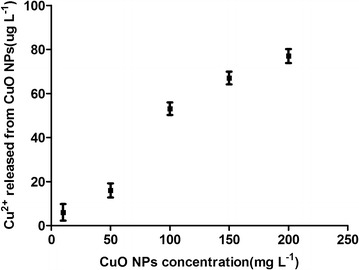
Cu^2+^ released from CuO NPs in culture media

The Cu^2+^ concentrations released from CuO NPs in culture media at 24 h are illustrated in Fig. 3. Only a small amount of Cu^2+^ was released in our experiment, which should be attributed to the aggregation of CuO in these culture media (Wang et al. 2013). In addition, the Cu^2+^ released from CuO NPs in culture media is also associated with the pH of the culture media and other environmental conditions of the experiment.

### Effects of CuO NPs, bulk CuO, and Cu^2+^ on the growth of *L. minor*

**Fig. 4:**
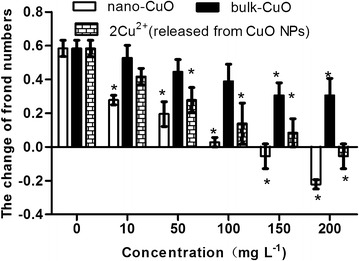
Frond number changes of *L. minor* in different culture media

The effects of CuO NPs, bulk CuO, and 2× concentration of Cu^2+^ released from CuO NPs on the frond number changes are shown in Fig. 4. The CuO NPs, bulk CuO, and Cu^2+^ showed a negative effect on frond number of *L. minor*. The CuO NPs showed the highest negative effect among the three kinds of material. When the CuO NPs concentration was 10 mg L^−1^, the frond number changes of *L. minor* decreased significantly compared with that of the control. The second highest negative effect on frond number changes was caused by 2× concentration of Cu^2+^, and the 2× Cu^2+^ released from 50 mg L^−1^ CuO NPs showed a significantly negative effect on the frond number changes of *L. minor*. Therefore, the negative effect of CuO NPs on frond number changes of *L. minor* was only partly due to the Cu^2+^ released by CuO NPs in the media.

**Fig. 5:**
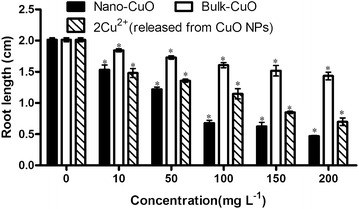
Root length of *L. minor* in different culture media

The effects of CuO NPs, bulk CuO, and twice concentration Cu^2+^ released from CuO NPs in culture media on the root length of *L. minor* are shown in Fig. 5. CuO NPs, bulk CuO, and 2× Cu^2+^ concentration from CuO NPs negatively affect the root length of *L.* *minor*. The adverse effect of CuO NPs on root length was the greatest among the three treatments, followed by negative effect of 2× Cu^2+^ concentration from CuO NPs. The three treatments showed significant effects on the root length of *L. minor* at ≥ 10 mg L^−1^. These factors significantly affected the root length of *L. minor* possibly because the plant-containing culture media were disturbed three times a day. The effect of CuO NPs on the micro-growth of another kind of duckweed (*Landoltia punctata*) and the uptake of Cu into plant tissue in comparison with a reference toxicant, CuCl_2_, have been studied (Gunawan et al. 2011), and CuO NPs are spontaneously synthesized in their study. Growth was inhibited (50 %) by a very low concentration of 1.0 mg L^−1^ CuO NPs after 96 h cultivation in petri dishes on a shaker. Such low concentration of CuO-NP can remarkably affect duckweed possibly because of the constant disturbance by the shaker. The roots of all kinds of duckweed are very tender, and thus, disturbance can easily harm their roots. The toxic materials can easily enter the root, resulting in significant damages to the root.

**Fig. 6:**
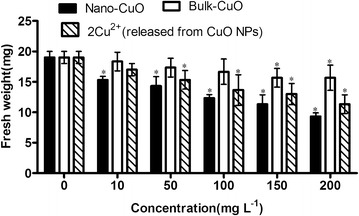
Fresh weight of *L. minor* in different culture media

The fresh weights of *L. minor* in different culture media are shown in Fig. 6. All of the three copper treatments showed negative effects on the fresh weight of *L. minor.* The negative effect of CuO NPs on fresh weight was the greatest among the three treatments, followed by 2× Cu^2+^ concentration released from CuO NPs. The plant was consisted in two parts, namely, the frond and root. The fresh weight of this plant consisted mainly of the frond, because the root of this kind of plant is very tender and light.

The micro-growth of *L. minor* indicated that CuO NPs exhibited greater effect on the growth of *L. minor* than the bulk CuO in the same concentration. the effect of CuO NPs on *L. minor* growth partly because of the Cu^2+^ releasing in culture media.

### Effects of CuO NPs, bulk CuO, and Cu^2+^ on the chlorophyll content of *L.minor* fronds

**Fig. 7:**
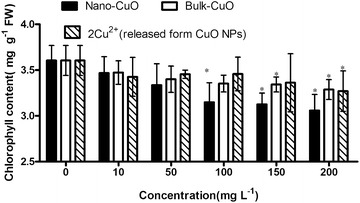
Chlorophyll content of *L. minor* frond in different culture media

The chlorophyll content of *L. minor* frond changed with the increase in concentrations of the three material treatments (Fig. 7). The chlorophyll content of *L.* *minor* decreased with the increase in concentration of CuO NPs, bulk CuO, and 2× Cu^2+^ concentration released from CuO NPs in culture media. In addition, the decrease in *L.* *minor* frond chlorophyll content was not as remarkable as the decrease in *L.* *minor* micro-growth. Based on visual inspection, the frond pigment did not change significantly with the increase in CuO NPs, bulk CuO, and 2× Cu^2+^ concentration released from CuO NPs. Different metallic oxide NPs show different effects on plant chlorophyll content. When the TiO_2_ NPs concentration increased in culture media, the frond of *L.* *minor* became dark green, with red color appearing inside the fronds in our previous study (Song et al. 2012). In the presence of sunlight, chlorophyll converts carbon dioxide and water into oxygen and glucose. The insignificant decrease in *L.* *minor* chlorophyll content in low CuO NP concentration media indicated that the oxygen and glucose synthesis proceeds normally in these culture conditions.

### Effects of CuO NPs, bulk CuO, and Cu^2+^ on antioxidant defense enzymes and MDA of *L. minor*

The effects of CuO NPs, bulk CuO, and twice concentration released from CuO NPs in culture media on the protective enzymes (i.e., POD, CAT, and SOD) of *L.* *minor* was also examined, as well as the MDA content. The production of active oxygen species is a biochemical change that possibly occurs when plants are subjected to harmful stress conditions. The chloroplasts and mitochondria of plant cells are important intracellular generators of reactive oxygen species (ROS). Internal O_2_ concentration is high during photosynthesis, and chloroplasts are particularly prone to generate ROS; therefore, these cytotoxic ROS can remarkably disrupt normal metabolism through oxidative damage of lipids, nucleic acids, and proteins. Deleterious effects of ROS and lipid peroxidation products are counteracted by an antioxidant defense system (Pejic´ et al. 2009). These damages can be examined by analyzing the changes of certain antioxidant enzymes, such as SOD, CAT, and POD.

**Fig. 8:**
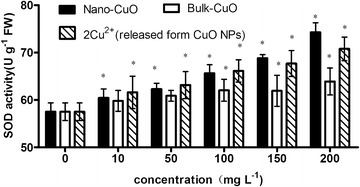
SOD activity of *L. minor* in different culture media

The SOD activity of *L.* *minor* increased with the increase in CuO NPs, bulk CuO, and 2× Cu^2+^ concentration released from CuO NPs in culture media (Fig. 8). The SOD activity of *L. minor* significantly increased from 10 mg L^−1^ CuO NP concentration in culture media compared with that of the control, and the SOD activity of *L. minor* was also significantly increased in 2× Cu^2+^ concentration released from 10 mg L^−1^ CuO NPs in culture media. The SOD activity of *L. minor* significantly increased until the bulk CuO concentration reached 100 mg L^−1^. SOD is an essential component of antioxidative defense system in plants. The enzyme is a major scavenger of O_2_^−^ and its enzymatic action results in the formation of H_2_O_2_ and O_2_. SOD performs a pivotal function in combating oxidative stress in plants, and a marked increase in SOD activity has been demonstrated to occur upon exposure to oxidative stress (Jalali-e-Emam et al. 2011). The plants in CuO NPs media accumulated O_2_^−^ in comparatively low concentration, and the release of Cu^2+^in culture media acts as a very important factor in SOD activity increase. *L.* *minor* accumulated a lower level of O_2_^−^ in bulk CuO media than in CuO NPs.

**Fig. 9:**
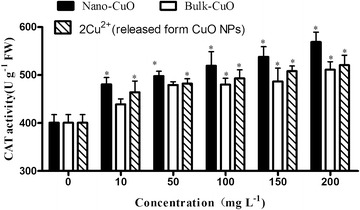
CAT activity of *L. minor* in different culture media

The CAT activity of *L. minor* increased with the increase in CuO NPs, bulk CuO, and 2× Cu^2+^ concentration released from CuO NPs (Fig. 9). The CAT activity of *L.* *minor* showed a significant increase at 10 mg L^−1^ CuO NP concentration and 2× Cu^2+^ concentration released from the same CuO NP concentration in culture media. Bulk CuO did not show significant effect on the CAT activity of *L.* *minor* until the bulk CuO concentration reached 100 mg L^−1^. CAT is one of the most important enzymes that scavenge ROS in plant cells. This enzyme participates in the main defense system against accumulation and toxicity of hydrogen peroxide and can function in controlling H_2_O_2_ level in cells. It acts on H_2_O_2_ and converts it to water and oxygen. CAT often shows the same trend as that of SOD when an organism is under stress. A study showed that SOD increases and CAT decreases when an organism is exposed to stress (Cui and Zhao 2011); however, some reports show that SOD decreases and CAT increases in organisms exposed to stress (Li et al. 2001; Sai Kachout et al. 2010). The activities of antioxidant defense enzymes are unstable and change with culture time. It is difficult to demonstrate if the plant can protect itself from environmental stress by using several antioxidant enzymes at a certain time. However, the activities of antioxidant defense enzymes often increase when the organism is under stress.

**Fig. 10:**
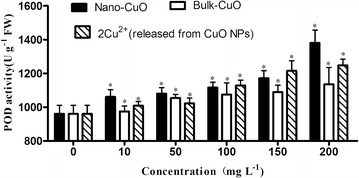
POD activity of *L. minor* in different culture media

The POD activity of *L. minor* increased with the increase of CuO NPs, bulk CuO, and 2× Cu^2+^ concentration released from CuO NPs (Fig. 10). The POD activity of *L.* *minor* significantly increased compared with the control from 10 mg L^−1^ concentration of the three kinds of materials. POD belongs to the group of enzymes involved in the growth, development, and senescence processes of plants. POD affects lignin and ethylene synthesis, as well as the decomposition of indole-3-acetic acid, and is involved in resistance against pathogens and wound healing. The POD activity of *L. minor* with increase in CuO NPs, bulk CuO, and 2× Cu^2+^ concentration released from CuO NPs indicates that the *L. minor* in CuO NPs, bulk CuO, and 2× Cu^2+^ concentration released from CuO NPs encountered oxidant stress in these experiment condition. The POD activity significantly increased in such low concentrations of the three condition which can be attributed to the wound of the root. As mentioned, plant roots exposed to these treatments can be easily broken with disturbance.

**Fig. 11:**
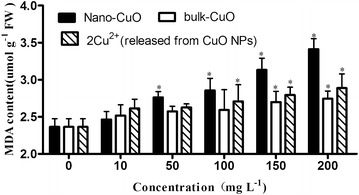
MDA content of *L. minor* in different culture media

The MDA content of *L.* *minor* increased with the increase of CuO NPs, bulk CuO, and 2× Cu^2+^ concentration released from CuO NPs in culture media (Fig. 11). The MDA content was significantly increased compared with that of the control from 50 mg L^−1^ CuO NP concentration in culture media. The MDA content of *L. minor* cultured in media with twice concentration of Cu^2+^ that released from CuO NPs increase in culture media and bulk CuO was also significantly different compared with that of the control in relative higher concentration. MDA is the decomposition product of polyunsaturated fatty acids of biomembranes, and its increase is a result of significant accumulation under high antioxidant stress. MDA content serves as an indicator of the extent of lipid peroxidation and is an indirect reflection of the extent of cell damage (Wang et al. 2011b). The significantly increased MDA content of *L.* *minor* demonstrated that the plant cells encountered serious damage under the culture conditions.

## Conclusions

Copper dioxide NPs aggregated in culture media. The stability of media with bulk CuO was higher than that of media with the same concentration of CuO NPs. CuO NPs released Cu^2+^ in culture media.

CuO NPs, bulk CuO, and Cu^2+^ decreased the growth of *L.* *minor*, and the effects of these three treatments on *L.* *minor* roots were more significant than the influence of the treatments on *L.* *minor* fronds. The effect of bulk CuO was not as remarkable as that of CuO NPs, and the effect of CuO NPs was partly due to the Cu^2+^ released from CuO NPs in the culture media.

*L. minor* cells exposed to CuO NPs accumulated more ROS compared with the plant cells exposed to the same concentration of bulk CuO. The plant cells accumulated ROS in CuO NP media partly because CuO NPs released Cu^2+^ in the culture media. The plant cell encountered serious damage when the CuO NP concentration was 50 mg L^−1^.
